# Influence of *ROBO1* and *RORA* on Risk of Age-Related Macular Degeneration Reveals Genetically Distinct Phenotypes in Disease Pathophysiology

**DOI:** 10.1371/journal.pone.0025775

**Published:** 2011-10-06

**Authors:** Gyungah Jun, Michael Nicolaou, Margaux A. Morrison, Jacqueline Buros, Denise J. Morgan, Monte J. Radeke, Yoshihiro Yonekawa, Evangelia E. Tsironi, Maria G. Kotoula, Fani Zacharaki, Nissa Mollema, Yang Yuan, Joan W. Miller, Neena B. Haider, Gregory S. Hageman, Ivana K. Kim, Debra A. Schaumberg, Lindsay A. Farrer, Margaret M. DeAngelis

**Affiliations:** 1 Medicine (Biomedical Genetics), Boston University Schools of Medicine and Public Health, Boston, Massachusetts, United States of America; 2 Ophthalmology, Boston University Schools of Medicine and Public Health, Boston, Massachusetts, United States of America; 3 Biostatistics, Boston University Schools of Medicine and Public Health, Boston, Massachusetts United States of America; 4 Neurology Boston University Schools of Medicine and Public Health, Boston, Massachusetts, United States of America; 5 Epidemiology, Boston University Schools of Medicine and Public Health, Boston, Massachusetts, United States of America; 6 Ophthalmology, Massachusetts Eye and Ear Infirmary, Boston, Massachusetts, United States of America; 7 Ophthalmology and Visual Sciences, John A. Moran Eye Center, Center for Translational Medicine, University of Utah, Salt Lake City, Utah, United States of America; 8 Center for the Study of Macular Degeneration, Neuroscience Research Institute, University of California Santa Barbara, Santa Barbara, California, United States of America; 9 Ophthalmology, Weill Cornell Medical College, New York, New York, United States of America; 10 Medicine, University of Thessaly, Larissa, Greece; 11 Genetics, Cell Biology, and Anatomy, University of Nebraska Medical Center, Omaha, Nebraska, United States of America; 12 Division of Preventive Medicine, Brigham and Women’s Hospital, Harvard Medical School, Boston, Massachusetts, United States of America; Rikagaku Kenkyūsho Brain Science Institute, Japan

## Abstract

*ROBO1* is a strong candidate gene for age-related macular degeneration (AMD) based upon its location under a linkage peak on chromosome 3p12, its expression pattern, and its purported function in a pathway that includes *RORA*, a gene previously associated with risk for neovascular AMD. Previously, we observed that expression of *ROBO1* and *RORA* is down-regulated among wet AMD cases, as compared to their unaffected siblings. Thus, we hypothesized that contribution of association signals in *ROBO1*, and interaction between these two genes may be important for both wet and dry AMD. We evaluated association of 19 single nucleotide polymorphisms (SNPs) in *ROBO1* with wet and dry stages of AMD in a sibling cohort and a Greek case-control cohort containing 491 wet AMD cases, 174 dry AMD cases and 411 controls. Association signals and interaction results were replicated in an independent prospective cohort (1070 controls, 164 wet AMD cases, 293 dry AMD cases). The most significantly associated *ROBO1* SNPs were rs1387665 under an additive model (meta P = 0.028) for wet AMD and rs9309833 under a recessive model (meta P = 6×10^−4^) for dry AMD. Further analyses revealed interaction between *ROBO1* rs9309833 and *RORA* rs8034864 for both wet and dry AMD (interaction P<0.05). These studies were further supported by whole transcriptome expression profile studies from 66 human donor eyes and chromatin immunoprecipitation assays from mouse retinas. These findings suggest that distinct *ROBO1* variants may influence the risk of wet and dry AMD, and the effects of *ROBO1* on AMD risk may be modulated by *RORA* variants.

## Introduction

Age-related macular degeneration (AMD) is a progressive retinal disease that severely reduces the quality of life. Unfortunately, medical treatments are limited particularly early in the course of disease before vision loss occurs. It is the most common cause of visual loss in the US with 10% prevalence in those 40 and older having any AMD and affecting more than 20 million people worldwide.[1] The disorder is more prevalent in whites than other ethnic groups, with almost eight-fold greater incidence than in African Americans for advanced AMD. The disease occurs equally in men and women.[1] There are two advanced clinical subtypes of AMD, non-exudative (geographic atrophy) and exudative (neovascular or wet). Although these advanced subtypes may have different pathophysiologic mechanisms, both can be preceded by the development of drusen (yellow-gray material in Bruch’s membrane) and retinal pigment epithelium (RPE) changes that progress into areas of atrophy, or in the case of wet AMD, the growth of new vessels from the choroid into the sub-RPE or sub-retinal space. It is this wet AMD, the neovascular subtype, that is responsible for loss of vision in the majority of cases. Therefore it is important to identify appropriate therapeutic targets either for prevention or treatment of early stages of AMD to reduce progression to these advanced stages.

Risk factors associated with AMD include family history,[2] white race,[1] smoking,[3], [4] and body mass index.[5] Prior studies have shown an inverse relationship between omega-3 fatty acids and the development of neovascular AMD.[6], [7] There is also evidence suggesting that hypertension and cataract surgery may increase the predisposition to AMD.[8] The existence of a genetic component to AMD was demonstrated initially by family studies and twin studies with heritability estimates ranging from 45–70%.[9] Two genetic variants, one in the complement factor H (*CFH*) gene[10]–[12] and the other in the *ARMS2/HTRA1* loci[13]–[16] have been consistently reported as major attributable risks for AMD. Association with a large number of additional loci, each with small effect, has been reported.[17]–[19] The products of many of these genes have a role in the complement system, cholesterol metabolism and protein transport.

*ROBO1* is a member of the immunoglobulin gene superfamily and is involved in axon guidance and neuronal precursor cell migration. *ROBO1* has three isoforms that are produced by alternative splicing. It is expressed in different tissues and organs including the retinal ganglion cell layer of the eye in mice and regulate the correct targeting of retinal ganglion cell axons along the entire visual projection.[20], [21] ROBO1 proteins are located on the cellular membrane and play a role in cell-adhesion.[22] Prior studies implicated *ROBO1* in ocular neovascularization via SLIT-ROBO1 signaling[23] and showed that inhibiting its expression in RPE cells resulted in suppression of proliferative vitreoretinopathy in animals.[24]–[26] Genetic association of *ROBO1* polymorphisms and AMD has not been reported thus far.

The chromosome 15q-linked *RORA* gene encodes the alpha retinoic acid receptor-related orphan receptor. It is associated with the development of the cerebellum and, together with *RORB*, with the maturation of photoreceptors in the retina. *RORA* has been implicated in the pathology of circadian rhythms, bone growth, angiogenesis, development of cones, cellular metabolism and a mediator in the immune and lipid metabolism pathways.[27] Linkage, association and expression studies have implicated *RORA* in AMD pathogenesis.[28]

Our previous expression study reported that *RORA* and *ROBO1* are down-regulated at least two-fold among affected individuals compared with their unaffected siblings.[28] In light of the involvement of *ROBO1* and *RORA* in eye development, specifically the retina, and our previous expression results, we investigated the association of AMD risk with *ROBO1* and the interaction of these two genes.

## Results

### Association of *ROBO1* SNPs with Wet and Dry AMD

The mean ages at exam in the New England Sibling Cohort (NESC) and an unrelated cohort from central Greece (GREEK) were comparable, but older by about 10 years than the mean age at diagnosis in the Nurses’ Health Study and Health Professionals Follow-up Study (NHS-HPFS) (Table 1). Because significant differences were seen between age and sex distribution among the three cohorts, all analyses included both these variables as covariates in order to control for their confounding effects. Analysis of linkage disequilibrium (LD) among *ROBO1* SNPs revealed a minimum of three distinct haplotype blocks (Figure S1): the first block encompassing the region between rs1387665 and rs4264688, the second between rs6548621 to rs9826366, and the third block including rs3923526, rs9309833, and rs7629503.

**Table 1:** Description of Datasets.

Study and Description		AMD		
		Controls	Wet AMD	Dry AMD
NESC				
	Total, N	198	352	106
	Average age at exam (SD)	75.40 (8.25)	73.80 (7.77)	76.65 (12.32)
	Gender (% of female)	56.1%	59.4%	65.1%
Greek				
	Total, N	213	139	68
	Average age at exam (years)	73.78 (7.25)	76.33 (7.49)	74.44 (7.99)
	Gender (% of female)	53.1%	58.8%	54.7%
NHS/HPFS				
	Total, N	1070	164	293
	Average age at exam (years)	60.21 (5.9)	61.07 (6.0)	59.53 (5.7)
	Gender (% of female)	63.6%	54.3%	70.7%

Abbreviations: SD, standard deviation; NESC, New England Sibling Cohort; Greek, central Greece cohort; NHS/HPFS, Nurses’ Health Study (NHS) and Health Professionals Follow-up Study (HPFS).

A total of 37 SNPs were identified in the discovery cohorts (for listing of SNPs see Table S1). Of these 37 SNPs, we focused on 19 tag SNPs, that reside upstream of the isoform *b* and in intron 3 of the isoform *a* in the human sequence (Table 2). We investigated association for neovascular (wet) form of AMD and dry AMD (Age Related Eye Disease Study [AREDS] category 2 and 3). In the NESC, five of the 19 *ROBO1* SNPs were associated with wet AMD at a nominal significance level at P<0.05 (Table 2). None of these SNPs were significantly associated with wet AMD in the GREEK cohort (P>0.05). Meta-analysis of the two cohorts revealed three SNPs from the middle LD block showed mild association (most significant SNP: rs7637338 with P = 0.021). The minor allele A of rs7637338 showed increased risk with an odds ratio (OR) of 1.39 (95% confidence interval [CI]  = 1.05–1.84). Three 5’ SNPs were moderately significant with dry AMD in the NESC, of which rs9309833 was the most significant (P = 0.005) (Table 3). Although these SNPs were not significant at P<0.05 in the GREEK cohort, the direction of effect was the same for each (Table 3) and the SNP rs9309833 remained significant in meta-analysis (meta P = 0.015). The two most significant SNPs for wet AMD (rs7637338) and for dry AMD (rs9309833) are uncorrelated (Figure S1) in both cohorts (r^2^<0.06), suggesting the possibility that these two signals are tagging independent causal variants in this gene.

**Table 2:** Association results of *ROBO1* SNPs for wet AMD in the NESC and GREEK cohorts, and in meta-analysis using an additive model.

SNP	Alleles	RA (RAF)	NESC		GREEK		Meta-Analysis	
			OR (95% CI)	P	OR (95% CI)	P	OR (95% CI)	P
rs1387665	G/A	A (0.52)	1.20 (0.94–1.53)	0.135	1.18 (0.84–1.66)	0.326	1.20 (0.98–1.46)	0.074
rs13076006	C/A	A (0.38)	0.76 (0.59–0.98)	0.036	1.03 (0.72–1.48)	0.867	0.84 (0.68–1.04)	0.105
rs4513416	T/C	T (0.38)	0.80 (0.63–1.03)	0.085	0.97 (0.69–1.38)	0.875	0.86 (0.70–1.05)	0.135
rs9810404	C/T	C (0.38)	0.79 (0.62–1.02)	0.068	1.02 (0.71–1.45)	0.934	0.86 (0.70–1.06)	0.150
rs7640053	C/A	C (0.38)	0.80 (0.62–1.02)	0.077	0.95 (0.67–1.35)	0.789	0.85 (0.69–1.04)	0.111
rs7615149	C/A	C (0.33)	0.79 (0.62–1.01)	0.060	1.04 (0.72–1.49)	0.850	0.86 (0.70–1.05)	0.148
rs7622888	C/T	C (0.32)	0.97 (0.74–1.27)	0.831	1.14 (0.77–1.71)	0.510	1.02 (0.82–1.28)	0.852
rs4264688	T/C	T (0.32)	0.99 (0.77–1.28)	0.949	1.15 (0.76–1.73)	0.518	1.03 (0.83–1.28)	0.778
rs6548621	A/G	A (0.42)	0.77 (0.61–0.97)	0.028	0.94 (0.66–1.33)	0.715	0.82 (0.67–0.99)	0.043
rs7622444	G/A	G (0.22)	1.44 (1.08–1.92)	0.013	1.05 (0.67–1.65)	0.819	1.32 (1.03–1.68)	0.026
rs9832405	A/G	A (0.41)	0.94 (0.75–1.19)	0.632	0.90 (0.61–1.34)	0.616	0.93 (0.76–1.14)	0.504
rs7637338	A/G	A (0.14)	1.31 (0.93–1.85)	0.125	1.56 (0.97–2.51)	0.068	1.39 (1.05–1.84)	0.021
rs6548625	C/T	C (0.34)	0.77 (0.60–0.99)	0.040	1.05 (0.74–1.50)	0.785	0.85 (0.70–1.05)	0.125
rs7623809	A/G	A (0.36)	0.78 (0.60–1.00)	0.054	1.04 (0.72–1.49)	0.840	0.86 (0.69–1.06)	0.146
rs4279056	G/A	G (0.38)	0.79 (0.62–1.02)	0.067	0.98 (0.69–1.39)	0.916	0.85 (0.69–1.04)	0.120
rs9826366	G/A	G (0.38)	0.81 (0.63–1.04)	0.099	0.96 (0.68–1.37)	0.843	0.86 (0.70–1.05)	0.144
rs3923526	T/A	T (0.16)	1.24 (0.91–1.70)	0.171	1.08 (0.70–1.66)	0.729	1.18 (0.92–1.53)	0.190
rs9309833	G/A	G (0.16)	1.43 (1.03–1.99)	0.035	0.95 (0.60–1.52)	0.838	1.25 (0.95–1.64)	0.108
rs7629503	T/G	T (0.27)	1.18 (0.90–1.53)	0.226	1.06 (0.73–1.53)	0.750	1.14 (0.92–1.41)	0.241

Alleles were provided from the plus (+) strand using the NCBI B36 assembly of dbSNP b126.

Abbreviations: SNP, Single Nucleotide Polymorphism; RA: reference allele used in association tests; RAF: reference allele frequency; OR: odds ratio; 95% CI: 95% confidence interval; P: P value.

**Table 3:** Association results of ROBO1 SNPs for dry AMD in the NESC and GREEK cohorts, and in meta-analysis using an additive model.

SNP	Alleles	RA (RAF)	NESC		GREEK		Meta-Analysis	
			OR (95% CI)	P	OR (95% CI)	P	OR (95% CI)	P
rs1387665	G/A	A (0.49)	0.94 (0.66–1.36)	0.749	1.07 (0.71–1.61)	0.747	1.00 (0.76–1.31)	0.981
rs13076006	C/A	C (0.41)	1.15 (0.79–1.68)	0.456	0.86 (0.56–1.34)	0.511	1.02 (0.77–1.36)	0.890
rs4513416	T/C	T (0.41)	1.17 (0.81–1.70)	0.400	0.96 (0.62–1.47)	0.838	1.08 (0.81–1.43)	0.614
rs9810404	C/T	C (0.41)	1.17 (0.81–1.70)	0.392	0.93 (0.60–1.43)	0.728	1.06 (0.80–1.41)	0.670
rs7640053	C/A	C (0.40)	1.16 (0.80–1.68)	0.447	0.80 (0.52–1.24)	0.322	0.99 (0.75–1.32)	0.949
rs7615149	C/A	C (0.35)	1.11 (0.77–1.60)	0.589	0.74 (0.47–1.18)	0.208	0.95 (0.71–1.26)	0.717
rs7622888	C/T	C (0.32)	1.06 (0.70–1.62)	0.780	1.27 (0.79–2.04)	0.322	1.15 (0.84–1.57)	0.386
rs4264688	T/C	T (0.31)	0.99 (0.64–1.51)	0.949	1.27 (0.79–2.04)	0.331	1.10 (0.80–1.52)	0.547
rs6548621	A/G	A (0.44)	0.92 (0.66–1.29)	0.633	1.00 (0.66–1.52)	0.992	0.95 (0.73–1.24)	0.716
rs7622444	G/A	G (0.20)	1.01 (0.63–1.62)	0.967	0.82 (0.46–1.46)	0.508	0.93 (0.65–1.34)	0.698
rs9832405	A/G	A (0.41)	0.90 (0.61–1.33)	0.591	1.49 (0.95–2.33)	0.085	1.11 (0.83–1.50)	0.470
rs7637338	A/G	A (0.12)	0.79 (0.44–1.44)	0.447	1.00 (0.50–1.98)	0.995	0.88 (0.56–1.37)	0.563
rs6548625	C/T	C (0.36)	1.00 (0.70–1.44)	0.990	0.75 (0.48–1.18)	0.212	0.89 (0.68–1.19)	0.440
rs7623809	A/G	A (0.38)	0.99 (0.68–1.45)	0.966	0.70 (0.44–1.11)	0.134	0.86 (0.64–1.16)	0.323
rs4279056	G/A	G (0.40)	1.03 (0.72–1.47)	0.878	0.72 (0.46–1.12)	0.142	0.89 (0.67–1.18)	0.421
rs9826366	G/A	G (0.40)	1.04 (0.72–1.49)	0.851	0.72 (0.46–1.12)	0.142	0.89 (0.68–1.18)	0.435
rs3923526	T/A	T (0.17)	1.73 (1.08–2.76)	0.023	1.22 (0.73–2.06)	0.447	1.48 (1.04–2.09)	0.028
rs9309833	G/A	G (0.17)	2.01 (1.24–3.27)	0.005	1.15 (0.69–1.94)	0.588	1.56 (1.09–2.22)	0.015
rs7629503	T/G	T (0.28)	1.75 (1.13–2.69)	0.011	1.05 (0.67–1.63)	0.831	1.36 (1.00–1.85)	0.050

Alleles were provided from the plus (+) strand using the NCBI B36 assembly of dbSNP b126.

Abbreviations: SNP, Single Nucleotide Polymorphism; RA: reference allele used in association tests; RAF: reference allele frequency; OR: odds ratio; 95% CI: 95% confidence interval; P: P value.

Our findings were extended to testing different genetic models with four SNPs covering each LD block and attempting to confirm the results in the NHS-NPFS replication cohort (Table 4). We confirmed association signals in the first block of *ROBO1* for wet AMD, with rs1387665 being the most significant under an additive model in meta-analysis of the three datasets (meta P = 0.028; OR = 1.18, CI = 1.02–1.37). However, this SNP was not associated with dry AMD (meta P>0.14). In contrast, rs9309833 from the third block was more strongly associated with dry AMD (meta P = 6×10^−4^; OR = 2.54, CI = 1.49–4.34) than with wet AMD (meta P = 0.047; OR = 1.88, CI = 0.99–3.56) under a recessive model. The association signal with rs9309833 for dry AMD remained significant even after adjusting for testing multiple SNPs, models, and traits (threshold P = 0.002 obtained with dividing 0.05 by 24 tests). There was no LD (r^2^ = 0) between rs1387665 and rs9309833 in all cohorts. These results suggest that there may be two or more independent causal variants residing in the different regions of the *ROBO1*, and the genetic models governing the effect of these variants may differ for wet and dry AMD.

**Table 4:** Association results of ROBO1 SNPs for wet AMD or dry AMD in meta-analysis under the three different genetic models (additive, dominant, and recessive) from the combined dataset including the NESC, the GREEK, and the NHS-HPFS cohort.

SNP	Model	RA	Wet AMD		Dry AMD	
			OR (95% CI)	P	OR (95% CI)	P
rs1387665	Add	A	1.18 (1.02–1.37)	0.0281	1.10 (0.95–1.28)	0.2179
	Dom		1.23 (0.96–1.58)	0.1027	1.21 (0.94–1.55)	0.1462
	Rec		1.28 (1.00–1.64)	0.0490	1.08 (0.84–1.38)	0.5413
rs4513416	Add	T	0.88 (0.75–1.02)	0.0979	0.93 (0.80–1.09)	0.3680
	Dom		0.81 (0.64–1.02)	0.0687	0.91 (0.73–1.14)	0.4212
	Rec		0.90 (0.67–1.19)	0.4486	0.91 (0.68–1.22)	0.5151
rs7622444	Add	G	1.11 (0.91–1.36)	0.2870	0.90 (0.73–1.11)	0.3093
	Dom		1.05 (0.83–1.32)	0.6948	0.82 (0.64–1.04)	0.0969
	Rec		1.74 (0.95–3.19)	0.0703	1.66 (0.91–3.02)	0.0993
rs9309833	Add	G	1.18 (0.96–1.44)	0.1150	1.33 (1.09–1.61)	0.0041
	Dom		1.13 (0.90–1.43)	0.3000	1.26 (1.01–1.59)	0.0451
	Rec		2.00 (1.01–3.96)	0.0465	2.54 (1.49–4.34)	6×10^−4^

Alleles were provided from the plus (+) strand using the NCBI B36 assembly of dbSNP b126.

Abbreviations: SNP, Single Nucleotide Polymorphism; RA: reference allele used in association tests; OR: odds ratio; 95% CI: 95% confidence interval; P: P value.

### Interaction between *ROBO1* and *RORA*

Four *ROBO1* tagging SNPs (rs1387665, rs4513416, rs7622444, and rs9309833) in a region that likely harbors alternative splice sites were selected for interaction analysis based on LD patterns in the region (Figure S1). Among the previously reported significant *RORA* SNPs for wet AMD (rs4335725 and rs8034864), haplotypes containing rs8034864 had the most consistent evidence of association in meta-analysis (Table 5). We therefore constructed additive models including one of four significant *ROBO1* SNPs, the *RORA* SNP (rs8034864), and an interaction term for the *ROBO1* and *RORA* SNPs. Other genetic models were not tested because of small sample sizes for many of the SNP×SNP genotype cells. Moderately significant interactions were found between *RORA* rs8034864 and *ROBO1* SNPs for both wet and dry AMD (Table 6). The interaction of rs8034864 and rs4513416 from the *ROBO1* gene remained significant (meta P for interaction  = 0.0042) after correction for testing eight interaction models (threshold P = 0.006). There was also significant evidence of interaction between *ROBO1* SNP rs9309833 and *RORA* SNP rs8034864 in affecting the risk of both wet (meta P for interaction  = 0.010) and early/intermediate dry AMD (meta P for interaction  = 0.037). The effect direction of these significant SNPs and the pattern of their interactions for early/intermediate dry AMD were consistent in all datasets (Table 6).

**Table 5:** Significant haplotypes in RORA for wet AMD in the NESC, GREEK, NHS-HPFS cohorts, and in meta-analysis using an additive model.

	NESC		GREEK		NHS-HPFS		Meta-Analysis	
Haplotype	OR (95% CI)	P	OR (95% CI)	P	OR (95% CI)	P	OR (95% CI)	P
rs8034864-rs730754	0.96 (0.56–1.67)	0.8959	1.36 (0.86–2.14)	0.1920	1.34 (1.01–1.79)	0.0417	1.28 (1.02–1.59)	0.0307
(T-G)								
rs8034864- rs12900948	0.65 (0.37–1.13)	0.1277	1.56 (0.95–2.56)	0.0819	1.52 (1.14–2.06)	0.0082	1.31 (1.03–1.66)	0.0260
(T-C)								

Abbreviations: OR: odds ratio; 95% CI: 95% confidence interval; P: P value.

**Table 6:** Summary of interaction analysis of ROBO1 SNPs (rs4513416, rs7640053, rs7622444 and rs9309833) and a RORA SNP (rs8034864) for wet and dry AMD in the three cohorts, NESC, GREEK, NHS-HPFS, and in meta-analysis.

ROBO1×RORA (Allele)		NESC		GREEK		NHS-HPFS		Meta-Analysis	
		OR (95% CI)	P	OR (95% CI)	P	OR (95% CI)	P	OR (95% CI)	P
Wet AMD:									
	rs1387665 (A)	1.18 (0.87–1.59)	0.2877	1.21 (0.74–1.97)	0.4556	1.20 (0.89–1.62)	0.2307	1.12 (0.92–1.37)	0.2414
	rs8034864 (T)	0.96 (0.54–1.70)	0.8938	1.61 (0.71–3.65)	0.2528	1.20 (0.74–1.95)	0.4660	0.99 (0.71–1.39)	0.9641
	INT	1.13 (0.72–1.75)	0.6001	0.54 (0.28–1.05)	0.0697	0.93 (0.63–1.39)	0.7368	1.12 (0.86–1.47)	0.4088
	rs4513416 (T)	0.86 (0.63–1.18)	0.3516	1.57 (0.94–2.64)	0.0846	0.90 (0.67–1.21)	0.4682	0.96 (0.79–1.17)	0.6807
	rs8034864 (T)	1.31 (0.83–2.08)	0.2514	2.13 (1.05–4.32)	0.0368	1.04 (0.67–1.61)	0.8604	1.28 (0.96–1.72)	0.0912
	INT	0.82 (0.51–1.32)	0.4162	0.45 (0.23–0.89)	0.0212	1.05 (0.71–1.55)	0.8126	0.84 (0.64–1.11)	0.2129
	rs7622444 (G)	1.58 (1.09–2.27)	0.0146	1.36 (0.70–2.67)	0.3684	0.70 (0.45–1.09)	0.1133	1.06 (0.82–1.37)	0.6541
	rs8034864 (T)	1.20 (0.82–1.74)	0.3449	1.02 (0.59–1.75)	0.9492	0.92 (0.64–1.32)	0.6580	1.03 (0.82–1.31)	0.7836
	INT	0.77 (0.47–1.26)	0.3062	0.56 (0.23–1.34)	0.1909	1.35 (0.74–2.46)	0.3222	1.07 (0.75–1.51)	0.7137
	rs9309833 (G)	2.21 (1.39–3.49)	7.2E–04	0.71 (0.30–1.69)	0.4372	1.16 (0.79–1.68)	0.4537	1.49 (1.13–1.97)	0.0046
	rs8034864 (T)	1.35 (0.97–1.87)	0.0788	0.73 (0.42–1.28)	0.2740	1.19 (0.84–1.70)	0.3333	1.29 (1.03–1.60)	0.0265
	**INT**	**0.48 (0.28**–**0.79)**	**0.0044**	**1.61 (0.58**–**4.48)**	**0.3615**	**0.87 (0.52**–**1.45)**	**0.5893**	**0.64 (0.45**–**0.90)**	**0.0102**
Dry AMD:									
	rs1387665 (A)	1.20 (0.78–1.86)	0.4047	0.66 (0.39–1.14)	0.1369	1.21 (0.96–1.51)	0.1023	1.24 (1.03–1.49)	0.0253
	rs8034864 (T)	1.50 (0.79–2.88)	0.2166	0.64 (0.30–1.39)	0.2598	1.05 (0.71–1.55)	0.7968	1.21 (0.89–1.64)	0.2177
	**INT**	**0.60 (0.35**–**1.04)**	**0.0672**	**2.09 (1.03**–**4.25)**	**0.0404**	**0.89 (0.65**–**1.22)**	**0.4732**	**0.75 (0.58**–**0.97)**	**0.0291**
	rs4513416 (T)	0.84 (0.53–1.33)	0.4548	0.63 (0.35–1.13)	0.1233	0.81 (0.64–1.02)	0.0682	0.79 (0.65–0.96)	0.0180
	rs8034864 (T)	0.57 (0.29–1.13)	0.1105	0.36 (0.15–0.86)	0.0217	0.79 (0.55–1.12)	0.1859	0.68 (0.51–0.91)	0.0101
	**INT**	**1.85 (1.08**–**3.19)**	**0.0260**	**2.30 (1.13**–**4.67)**	**0.0212**	**1.22 (0.89**–**1.67)**	**0.2198**	**1.45 (1.12**–**1.87)**	**0.0042**
	rs7622444 (G)	1.24 (0.72–2.15)	0.4339	1.68 (0.80–3.52)	0.1722	0.89 (0.65–1.21)	0.4537	0.91 (0.71–1.17)	0.4733
	rs8034864 (T)	1.17 (0.72–1.89)	0.5290	1.61 (0.84–3.10)	0.1507	0.95 (0.72–1.25)	0.6964	0.94 (0.75–1.18)	0.6037
	INT	0.59 (0.29–1.18)	0.1339	0.50 (0.19–1.33)	0.1657	0.97 (0.61–1.54)	0.8958	0.93 (0.65–1.34)	0.7140
	rs9309833 (G)	3.67 (1.99–6.78)	3×10–^5^	1.11 (0.47–2.61)	0.8165	1.38 (1.04–1.82)	0.0248	1.55 (1.22–1.98)	4×10^−4^
	rs8034864 (T)	1.37 (0.83–2.26)	0.2112	1.57 (0.83–2.98)	0.1681	1.06 (0.80–1.41)	0.6940	1.05 (0.83–1.32)	0.6885
	**INT**	**0.36 (0.18**–**0.73)**	**0.0043**	**0.62 (0.23**–**1.69)**	**0.3501**	**0.77 (0.51**–**1.15)**	**0.2011**	**0.70 (0.50**–**0.98)**	**0.0367**

Alleles were provided from the plus (+) strand using the NCBI B36 assembly of dbSNP b126. Bold cells represent nominally significant association with P<0.05.

Abbreviations: OR: odds ratio; 95% CI: 95% confidence interval; P: P value; INT: interaction term

**Figure 1:**
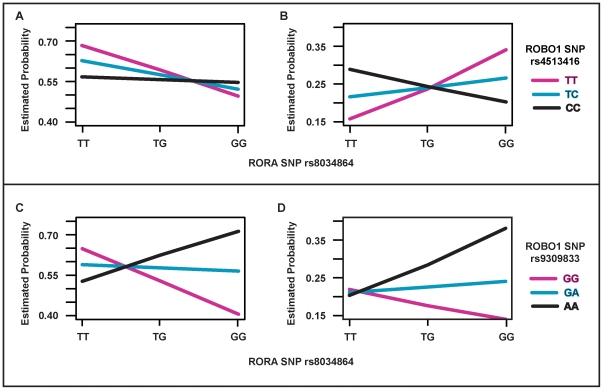
Estimated probabilities for different categories of genotypes between *ROBO1* SNPs and a *RORA* SNP in meta-analysis. X-axis is the categories of genotypes for rs8034864 from the *RORA* gene, and Y-axis is the estimated probabilities of different genotypic groups for rs4513416 (A and B) and rs9309833 (B and C) from the *ROBO1* gene after adjusting for covariates. Graphs for wet AMD are shown in A and C, and for dry AMD in B and D.

Analysis of the full logistic models (Fig. 1) revealed that comparing with the dosage effect of the rs4513416 *C* allele for wet AMD (Fig. 1A) that for early/intermediate dry AMD was modulated by the dose of the rs8034864 *T* allele (Fig. 1B). Interaction between *ROBO1* SNP rs9309833 and *RORA* SNP rs8034864 was significant for both wet (Fig. 1C) and early/intermediate dry AMD (Fig. 1D) such that risk of AMD increased according to dose of the rs8034864 *G* allele among rs9309833 *AA* homozygotes, whereas AMD risk decreased according to dose of the rs8034864 *G* allele among rs9309833 *GG* homozygotes.

### Chromatin Immunoprecipitation (ChIP) Assays

**Figure 2:**
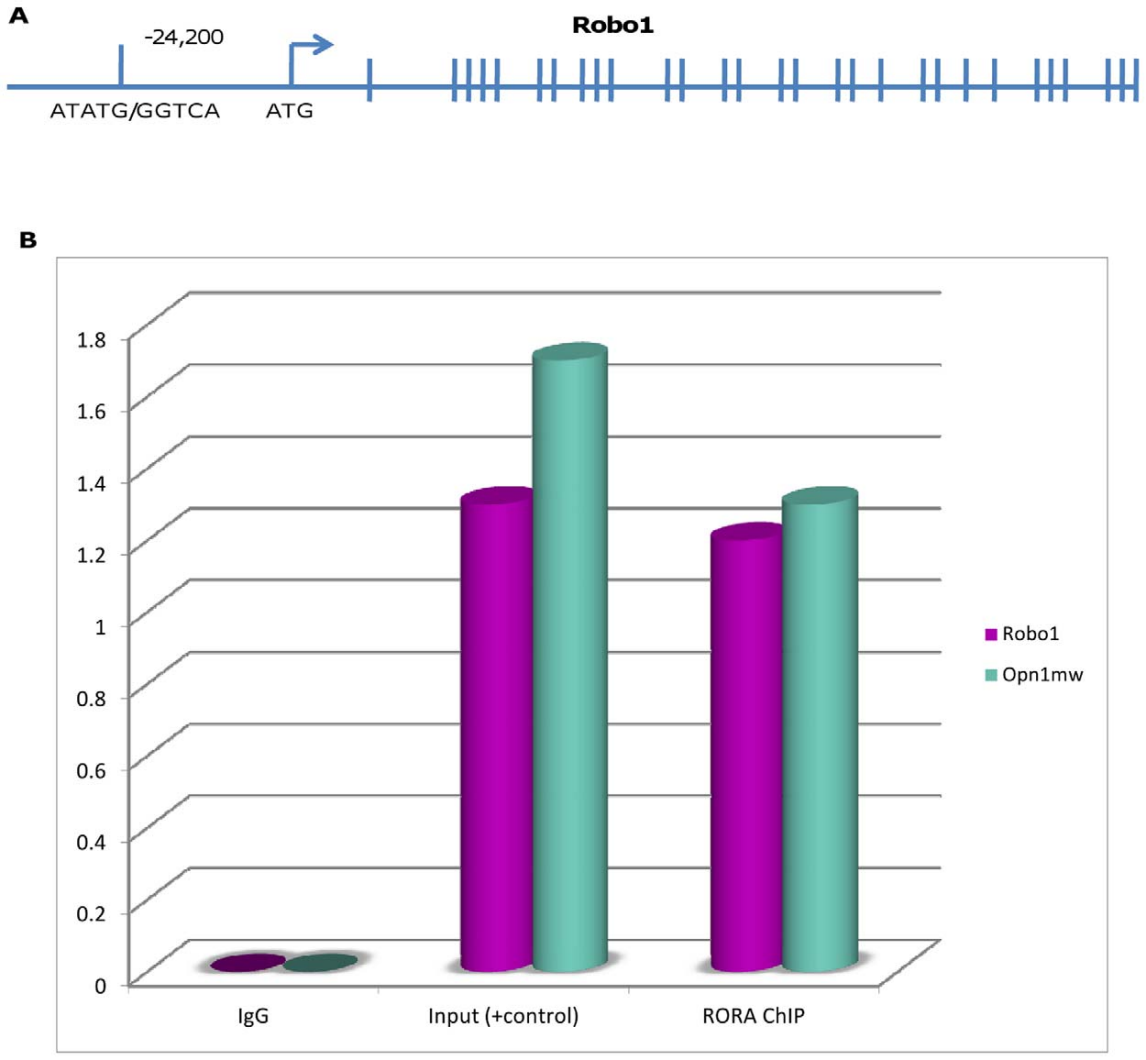
*RORA* binding upstream to regulatory region of *ROBO1*. **A.** Schematic of *ROBO1* gene and approximate location of *RORA* RE binding site. **B.** qRT-PCR of ChIP samples using normal, adult, mouse retina identifies Robo1 as a target of Rora. Opn1mw was previously reported as a target of RORA and is therefore a positive control. Neither Robo1 nor Opn1mw amplified out of the IgG ChIP (negative control).

The protein encoded by the *RORA* gene is known to bind to response elements of several genes to enhance the expression of those genes. We conducted an experiment to test whether the *RORA* gene product (Rora) binds to regulatory sequence elements of the *ROBO1* gene to determine if Rora directly binds to and can regulate Robo1 gene expression in vivo. A recent report suggested that the Rora putative response element recognition sequence is RGGTCA where R represents any purines.[27] We evaluated approximately 30 kilobases (kb) of Robo1 5’ untranslated regulatory region and also intron 1 to identify a putative Rora binding site in mouse. A potential binding site consisting of ATATG[GGTCA] 24,200 bp from the Robo1 start codon was identified (Fig. 2A). This binding site (at base pair position 79,091,190 in human) corresponds to a site 338,621 base pairs downstream of the first significantly associated *ROBO1* promoter SNP, rs1387665.

Rora binding sequences were precipitated and isolated from normal (C57BL6/J) mouse retinas. Rora binding to Robo1 regulatory region was determined by quantitative real time PCR (Fig. 2B). The green opsin (Opn1mw) locus control region was previously reported as a Rora binding site and therefore served as a positive control.[29] We confirmed the binding affinity between Rora and Robo1 regulatory region with similar strength compared with that with the positive control (Fig. 2B). The absence of amplification from IgG precipitated samples demonstrated the specificity of the Rora antibody and validated the binding of Rora to the Robo1 response element sequence.

### Gene Expression Profiling in Human Donor Eyes

**Figure 3:**
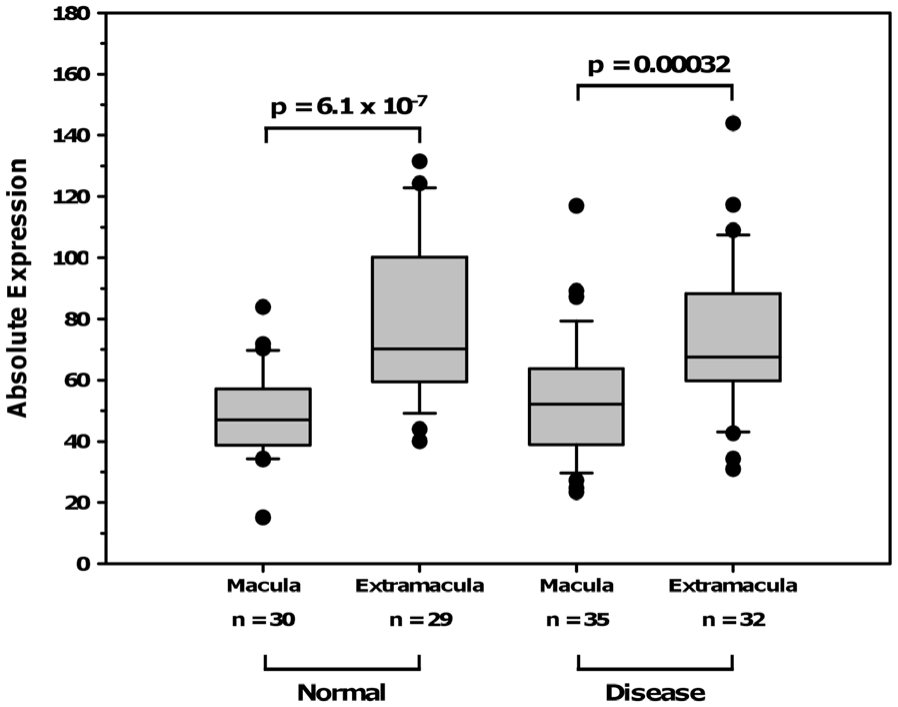
Results from gene expression studies in *ROBO1*. Absolute expression of *ROBO1* in the RPE-Choroid is plotted on the Y-axis, and values for the macula and extra macula are plotted for both normal eyes and eyes with all AMD subtypes.

Expression of both *ROBO1* and *RORA* was detected in the RPE-choroid and the retina. Of the genes examined in a whole transcriptome analysis of ocular tissues derived from 66 human donors, no significant association as a function of age was observed. We did not observe statistically significant differences in *RORA* expression (data not shown), but *ROBO1* expression was significantly different between the macula and extramacula in both normal and AMD RPE-choroid (Fig. 3). This complements our previous finding in immortalized cell lines, which showed *ROBO1* had decreased expression by at least two fold in index patients with neovascular AMD compared to their unaffected siblings.[28]

## Discussion

We demonstrated significant association with *ROBO1* SNPs showing increased risk of wet and early/intermediate dry AMD in a combined cohort of sibling pairs, cases and controls from Central Greece, as well as a prospective case control study from the NHS/HPFS. Moreover, we discovered variants from different LD blocks that could explain the separate association signals for wet and early/intermediate dry AMD. This suggests that different regions of this gene may be responsible for risk of the different subtypes of AMD or possibly indicate who may progress to wet or neovascular AMD, which would have implications for therapeutic targets. Previous genetic association studies reported that *ROBO1* polymorphisms are associated with other diseases of complex etiology. For example, variation in *ROBO1* is associated with language ability[30] and shared genetic factors between asthma and obesity in children.[31] A prior study shows that *ROBO1* is also associated with autism, showing that mRNA expression is significantly down-regulated in those with autism.[32] Furthermore, a role for Robo1 expression in retinal angiogenesis has been demonstrated in a rabbit model of proliferative retinopathy as well as in vitro studies of epiretinal and subretinal membranes from patients with proliferative retinopathies.[25]

In addition to establishing association of *ROBO1* with AMD, we were able to document a statistically significant interaction between *ROBO1* and another AMD-associated gene, *RORA*. *RORA,* a gene that is known to be involved in wet AMD based on retrospective[28] and prospective[33] studies, regulates expression of genes in the mammalian clock mechanism[34] and in lipid metabolism by changing levels of total plasma cholesterol, triglycerides, and apolipoprotein.[35] DNA response elements of *RORA* comprise a 5’ AT-rich sequence and along with coactivators, change constitutive activation of target gene transcription.[34] Interestingly, analogous to *ROBO1*, the protein product of the *RORA* gene is also significantly reduced in the autistic brain.[36] Statistical association and interaction with *ROBO1* and *RORA* genes were validated by a bioinformatic search for response elements residing on *ROBO1* sequences followed by experimental confirmation using chromatin immunoprecipitation assays and qRT-PCR in normal mouse maculae. Using the known Rora response elements as a positive control, we established similar quantity of precipitation with the regulatory region of the *ROBO1* gene as a potential Rora binding site. This gave further evidence underlying a biological interaction between a Rora product and the regulatory element of the *ROBO1* gene. We are currently extending the findings in mouse using chromatin immunoprecipitation assays to direct sequencing in human donor eyes and immortalized patient cell lines.

Our previous observation of down-regulation for *ROBO1* in immortalized cell lines[28] was validated in human donor RPE-choroid and retina in the current study. Similar to the reduced expression of this gene in autism, expression levels for *ROBO1* in AMD macular and peripheral retinas compared with normal maculas and peripheral retinas were significantly reduced. This also lends support for the hypothesis that AMD is a systemic disease with a localized manifestation, as significant differences in expression of *ROBO1* on a systemic level, in cell lines,[28] was confirmed on the DNA level although no differences in expression of *ROBO1* were seen between AMD eyes and non-AMD eyes. Our findings along previous reports suggest that pathogenesis of *ROBO1* and *RORA* in complex diseases is potentially shared by down-regulation of expression in response to neurodegeneration and these findings could have significant implications for therapeutic interventions and drug delivery.

Our statistical findings along with molecular verification have improved our understanding on the potential synergetic effect of *ROBO1* and *RORA* in the early/intermediate AMD stages as well as the severe advanced neovascular form of AMD. In addition to the discovery of multiple variants in *ROBO1* that may differentiate wet and early/intermediate dry AMD, SNPs in *ROBO1* were found to interact with *RORA* in the early/intermediate dry form of AMD in meta-analysis that were not found to significantly interact in the neovascular AMD subtype as shown in Table 6.

The study design is unique from others such that we separated two subtypes of AMD from all AMD or advanced AMD, to investigate multiple variants that may be involved in the early/intermediate or advanced/severe neovascular AMD subtype. This approach is supported by an illustration of a review[37] that three different components of AMD, drusen formation, neovascularization, and RPE atrophy, have seen in many different complex diseases, implying that there may be independent underlying mechanisms to develop each of these components. A previous study also demonstrated that drusen formation may have both unique and shared underlying genetic mechanisms with intermediate or advanced AMD development.[38] Specifically, this study showed that drusen formation as an intermediate stage of advanced AMD types identified previously known linkage signals for advanced AMD as well as novel peaks. One of the unique peaks for large drusen size is on chromosome 19q13.21 that is accounted for the genotype of *APOE* gene. These further support our results on differential association signals for wet and early/intermediate dry AMD. Our hypothesis-driven, genomic convergent approach based on prior biological plausibility provided collective evidence from statistical tests and molecular experiments demonstrating potentially yet another pathway underlying AMD pathogenesis. Thus, our results, together with statistical findings and molecular verification, warrant further investigation for both diagnostics and therapeutics implications by taking both genes into consideration, as they appear to work together.

## Methods

### Ethics Statement

This study was reviewed and approved by the Institutional Review Boards (IRBs) at Massachusetts Eye and Ear Infirmary, the University of Utah, and Boston University and conforms to the tenets of the Declaration of Helsinki. Written informed consent was obtained from all participants.

### Subjects and Phenotypes

Our study comprised two discovery cohorts, the New England Sibling Cohort (NESC) that has 1,011 individuals including 500 sibpairs and an unrelated cohort from central Greece with 344 unrelated subjects (GREEK). Replication of findings from this sample was sought in a cohort of 1,528 unrelated subjects from the Nurses’ Health Study and Health Professionals Follow-up Study (NHS-HPFS). Details of recruitment, diagnostic criteria and subject classification for the NESC are described elsewhere.[28], [39] In brief, at least one individual from each family had the neovascular (wet) form of AMD in at least one eye after excluding patients with a retinal pigment epithelium detachment, myopia, ocular histoplasmosis syndrome, angioid streaks, choroidal rupture, any hereditary retinal diseases other than AMD, and previous laser treatment for retinal conditions other than AMD. A total of 352 wet AMD probands, 106 early/intermediate dry probands (Age Related Eye Disease Study [AREDS] category 2 and 3), and 198 normal siblings from 284 families comprising 352 wet AMD sibpairs and 76 early/intermediate dry sibpairs were available for this study. All but 87 of the sibpairs were discordant for AMD. The GREEK cohort was enrolled at the University Hospital of Larissa outpatient medical clinics in central Greece. The diagnosis of AMD in this cohort was confirmed by optical coherence tomography and Fluorescein angiography.[28], [39] A total of 139 wet AMD cases, 68 early and intermediate dry AMD cases, and 213 controls with normal macula were available after excluding patients with geographic atrophy. The NHS-HPFS comprised 1,070 controls, 164 wet AMD cases, and 293 dry AMD cases. We used two different definitions for affection status, wet AMD and dry AMD, after excluding patients with geographic atrophy[33].

### Genotyping

Initially, genotyping was performed with tagging single nucleotide polymorphisms (SNPs) from the *ROBO1* gene. To assess variation within this gene, tag SNPs were chosen to span the *ROBO1* gene using data from the HapMap (http://www.hapmap.org/) after applying for the following criteria: 1) minor allele frequency was greater than 10%, 2) linkage disequilibrium (LD; r^2^) was at least 0.8, and 3) tagged for at least 6 other SNPs. These SNPs were genotyped using a combination of Sequenom and TaqMan. For the SNPs genotyped via Sequenom, multiplex PCR assays were designed using Sequenom SpectroDESIGNER software (version 3.0.0.3) (Sequenom, San Diego, CA) by inputting sequence containing the SNP site and 100 base pair (bp) of flanking sequence on either side of the SNP. Briefly, 10 ng of genomic DNA was amplified in a 5 uL reaction containing 1X HotStar Taq PCR buffer (Qiagen, Valencia, CA), 1.625 mM MgCl2, 500 uM each dNTP, 100 nM each PCR primer, 0.5 U HotStar Taq (Qiagen). The reaction was incubated at 94°C for 15 minutes followed by 45 cycles of 94°C for 20 seconds, 56°C for 30 seconds, 72°C for 1 minute, followed by 3 minutes at 72°C. Excess dNTPs were then removed from the reaction by incubation with 0.3 U shrimp alkaline phosphatase (USB, Cleveland, OH) at 37°C for 40 minutes followed by 5 minutes at 85°C to deactivate the enzyme. Single primer extension over the SNP was carried out in a final concentration of between 0.625 uM and 1.5 uM for each extension primer (depending on the mass of the probe), iPLEX termination mix (Sequenom) and 1.35 U iPLEX enzyme (Sequenom) and cycled using a two-step 200 short cycles program; 94°C for 30 seconds followed by 40 cycles of 94°C for 5 seconds, 5 cycles of 52°C for 5 seconds, and 80°C for 5 seconds, then 72°C for 3 minutes. The reaction was then desalted by addition of 6 mg cation exchange resin followed by mixing and centrifugation to settle the contents of the tube. The extension product was then spotted onto a 384 well SpectroCHIP before being flown in the MALDI-TOF mass spectrometer. Data was collected, real time, using SpectroTYPER Analyzer 3.3.0.15, SpectraAQUIRE 3.3.1.1 and SpectroCALLER 3.3.0.14 (Sequenom). Additionally, to ensure data quality, genotypes for each subject was also checked manually. For the SNPs genotyped via TaqMan, either TaqMan Pre-Designed SNP Genotyping Assays or Custom TaqMan SNP Genotyping Assays (Applied Biosystems) kits were ordered (for listing of SNPs and probes, see Table S2). The 40X stock of the probes were diluted to 16X with 0.5X tris-EDTA and stored at −20°C. The amplification reaction was carried out in a total reaction volume of 16.25 µL containing 2.5 µL DNA (10ng), 1.25 µL of 16X probe, and 12.5 µL of TaqMan Genotyping Master Mix. Sample DNA was amplified using the following reaction: 1 min at 60°C, 10 min at 95°C, and 40 cycles of 15 sec. at 92°C and 1 min at 60°C. The amplification reaction is carried out on thermocyclers and then fluorescence is measured on the ABI 7500 Real-Time PCR System by which the genotypes are analyzed with the accompanying software, or, in some cases, manually.

All genotyped SNPs met quality control thresholds of call rate of at least 90% and being in Hardy-Weinberg equilibrium (HWE) (P>0.01). LD among *ROBO1* SNPs was evaluated using the HapMap CEU reference population. At least one SNP from each haplotype block, delineated on the basis of pairwise estimates of LD (r^2^) >0.5, was further analyzed under different genetic models and in the interaction analyses. This SNP selection scheme both sufficiently accounts for the potential contribution of *ROBO1* individually and through interaction with *RORA* to AMD risk and minimizes the penalty of multiple testing.

### Sequencing

Based on the location of the significant SNPs found in the initial screen of *ROBO1*, direct sequencing was also performed on the promoter and exons 1, 2, and 3 in order to discover novel variation. For these reactions, oligonucleotide primers were selected using the Primer3 program http://primer3.sourceforge.net/) to encompass the SNP and flanking intronic sequences. All PCR assays were performed using genomic DNA fragments from 20 ng of leukocyte DNA in a solution of 10 PCR buffer containing 25 mM of MgCl2, 0.2 mM each of dATP, dTTP, dGTP, and dCTP, and 0.5 U of Taq DNA polymerase (USB Corporation). Five molar betaine was added to the reaction mix for rs2414687 (Sigma–Aldrich, St. Louis, MO). The temperatures used during the polymerase chain reaction were as follows: 95°C for 5 min followed by 35 cycles of 58°C for 30 s, 72°C for 30 s and 95°C for 30 s, with a final annealing at 58°C for 1.5 min and extension of 72°C for 5 min. For sequencing reactions, PCR products were digested according to manufacturer’s protocol with ExoSAP-IT (USB Corporation) then were subjected to a cycle sequencing reaction using the Big Dye Terminator v 3.1 Cycle Sequencing kit (Applied Biosystems, Foster City, CA) according to manufacturer’s protocol. Products were purified with Performa DTR Ultra 96-well plates (Edge Biosystems, Gaithersburg, MD) in order to remove excess dye terminators. Samples were sequenced on an ABI Prism 3100 DNA sequencer (Applied Biosystems). Electropherograms generated from the ABI Prism 3100 were analyzed using the Lasergene DNA and protein analysis software (DNASTAR, Inc., Madison, WI). Electropherograms were read independently by two evaluators without knowledge of the subject’s disease status. All patients were sequenced in the forward direction (5’–3’), unless variants or polymorphisms were identified, in which case confirmation was obtained in some cases by sequencing in the reverse direction.

### Statistical Analysis

Linkage disequilibrium (LD) among the genotyped SNPs was determined using Haploview (version 4.2; http://www.broadinstitute.org/scientific-community/science/programs/medical-and-population-genetics/haploview/haploview). *ROBO1* SNPs were tested for association with wet and dry AMD classification groups in the discovery cohorts using a logistic regression approach under an additive model including age and sex as covariates. Generalized Estimating Equations (GEE) were used in the analysis of the family dataset to account for familial correlations[40] and a generalized linear model approach was used for the unrelated cohorts. All analyses were performed using the R package (R2.2.1; http://www.r-project.org/). Haplotype analysis was performed using UNPHASED (version 3.1.4; http://homepages.lshtm.ac.uk/frankdudbridge/software/unphased/)[41], [42] which can account for family- based data. Association results obtained from individual datasets were combined by meta-analysis using the inverse variance method implemented in the software package METAL (http://www.sph.umich.edu/csg/abecasis/Metal/).[43] Effect sizes were determined by summing the regression coefficients weighted by the inverse variance of the coefficients. Significant findings from the combined discovery cohorts were evaluated for association in the replication sample. Results from the three cohorts were combined by meta-analysis. SNPs with nominally significant P values (< 0.05) in the combined sample (meta P) were further tested under dominant and recessive models.

Four nominally significant SNPs (meta P<0.05) from the *ROBO1* gene were selected for interaction analysis. Association of *RORA* SNPs for wet AMD was confirmed using haplotype analysis using the UNPHASED program. One *RORA* SNP (rs8034864) was selected from haplotype analysis results for tests of interaction with *ROBO1*. Interaction of each of four *ROBO1* SNPs with a *RORA* SNP was assessed by comparing a baseline additive model, which includes an independent term for each SNP, to the full additive model which includes the SNP main effects plus an interaction term. Significant findings in the discovery datasets were tested for confirmation in the NHS-HPFS cohort. Using the estimates from the meta-analysis, probabilities from a full logistic model, P_h_(X)  = 1/{1+exp[-(α+β_1_SNP_1_+ β_2_SNP_2_+ β_3_SNP_1_ xSNP_2_)]}, under the assumption of the same age and sex was calculated for each genotypic categories for wet and dry AMD and plotted against grouped genotypes from the two interacting SNPs.

### Chromatin Immunoprecipitation and Quantitative (real time) RT-PCR

Total RNA was isolated from P30.5 eyes (n = 8) from B6 mice using Trizol for quantitative real time—PCR (qRT-PCR). Sample preparation, qRT-PCR reaction and analysis were performed as described previously.[44] Chromatin immunoprecipitation (ChIP) was performed on P30 retinal lysates from B6 mice as described previously.[44] Briefly, immunoprecipitation was performed overnight with 4ug of Rora antibody (goat, Santa Cruz Biotechnology) and immunoglobulin (Ig) antibody (goat, R&D Systems). Quantitative RT-PCR was performed using 4ul of Rora, 4ul of IgG, and 2ul of input using real-time conditions described previously.[29] Tested genes included the “locus control region” (response site) of Opn1mw and the response site on Robo1. Opn1mw-LCR was used as a positive control as previously published [29] and is located 3,896bp from the start codon. An approximately 200 base pair region surrounding the Robo 1 response element sequence region was amplified using primers: Robo1F 3' CATTTGGACCTTGTGTGTCT 5', Robo1R: 3' GTCTCTGCCACAATCTCACT 5'. To map this mouse variation to the corresponding sequence in human, Ensembl genomic alignments were used: for mouse, Robo1 ENSMUSG00000022883 and for human, ROBO1 ENSG00000169855 was used.

### RPE-Choroid Expression Profile Assessment

Whole transcriptome expression profiles were obtained from 126 RPE-choroid and 118 retina punches (each 6mm in diameter) obtained from the macular and extramacular regions of eyes from 66 human donors. These eyes were selected from a well-characterized repository including 3,903 donors collected over a 20 year period at the University of Iowa and St. Louis University by Dr. Hageman. Medical and ophthalmic histories, a family questionnaire, blood, and sera, were obtained from the majority of donors. Gross pathologic features, as well as the corresponding fundus photographs and angiograms, when available, of all eyes in this repository were read and classified by retinal specialists. Fundi and/or posterior poles were graded using a slightly modified version of two standardized classification systems, as published previously.[45]-[49] The ages of the donors ranged from 9 to 101 years; approximately 50% had documented clinical histories of AMD. RNA expression profiles were assessed using two-color, 44K Agilent Whole Genome *in situ* oligonucleotide microarray analysis and a universal reference RNA experimental design. The universal reference RNA consisted of a 1:1 pool of RPE-choroid and retina RNA generated from donors with and without AMD. After correcting for dye effects using LOWESS normalization, the net intensity values were determined and expressed as a percentage of the total array intensity. The ratios of the experimental and reference RNA signals were calculated, and then the normalized percent total of each experimental value was calculated by multiplication using the geometric mean of all determinations of each probe’s reference RNA value. For those probes with replicates in the array, the average values were determined. Inter-array differences were further corrected by quantile normalization and probes that did not have net intensities values greater than six times the standard deviation of the background in at least 5% of the samples were omitted. This resulted in a final data set comprised of 28,127 unique probes. Expression of the *ROBO1* and *RORA* genes was examined.

## Supporting Information

Figure S1 Linkage disequilibrium (r^2^) between SNPs from the ROBO1 gene for wet or dry AMD in NESC (A) and in GREEK (B) cohort.
(TIF)

Table S1 **List of** ***ROBO1*** **SNPs investigated.**
(DOCX)

Table S2 ***ROBO1*** **TaqMan Probes.**
(DOCX)
