# Supplementary figures and images for: Influence of ROBO1 and RORA on Risk of Age-Related Macular Degeneration Reveals Genetically Distinct Phenotypes in Disease Pathophysiology
Source: PLoS One. 2011 Oct 6;6(10):e25775. doi: 10.1371/journal.pone.0025775 (PMC3188561; doi:10.1371/journal.pone.0025775)

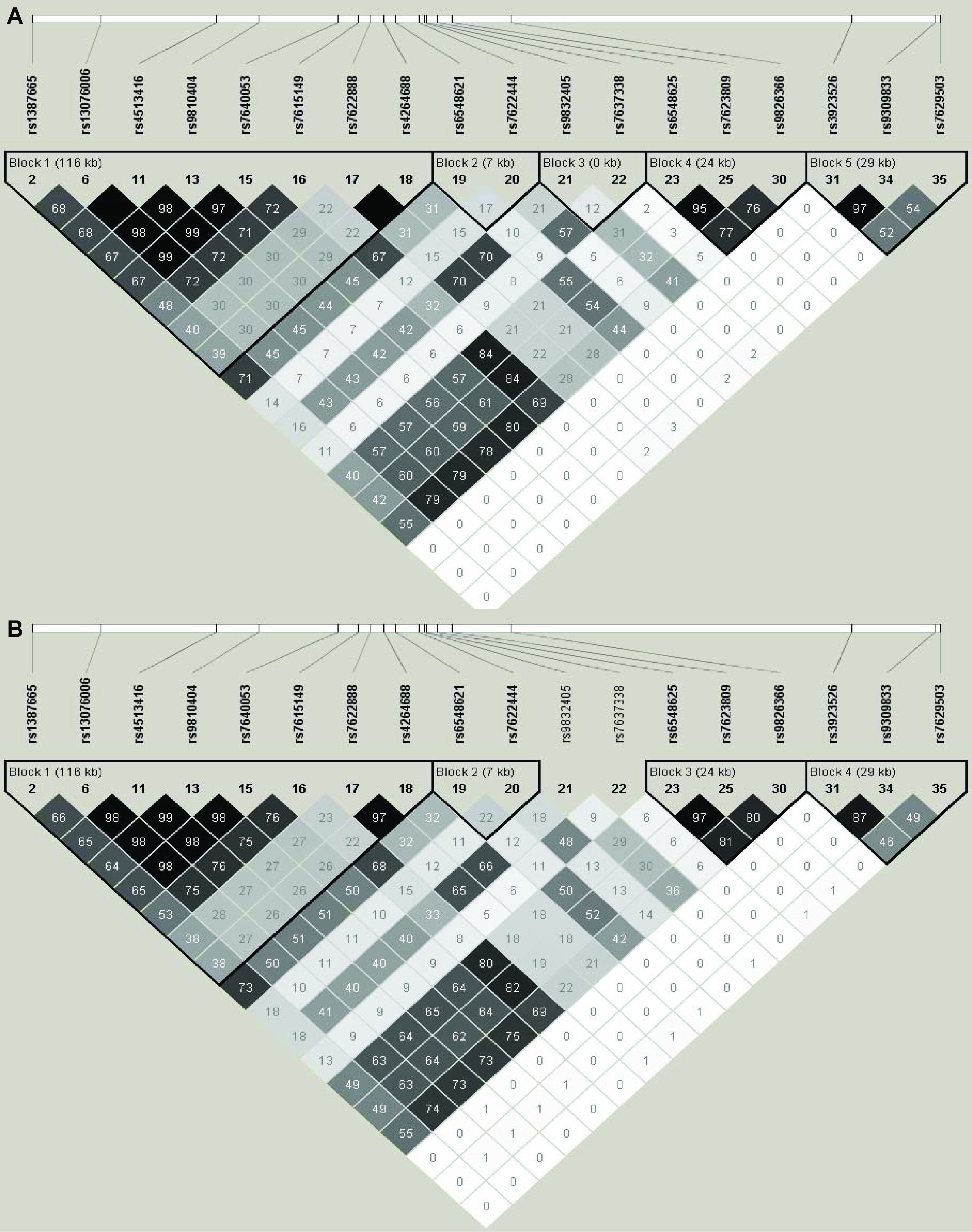

Supplement: Figure S1 — Linkage disequilibrium (r2) between SNPs from the ROBO1 gene for wet or dry AMD in NESC (A) and in GREEK (B) cohort. (TIF) [file pone.0025775.s001.tif]
