# Supplementary material for: Influence of ROBO1 and RORA on Risk of Age-Related Macular Degeneration Reveals Genetically Distinct Phenotypes in Disease Pathophysiology
Source: PLoS One. 2011 Oct 6;6(10):e25775. doi: 10.1371/journal.pone.0025775 (PMC3188561; doi:10.1371/journal.pone.0025775)
Supplement: Table S1 — List of ROBO1 SNPs investigated. (DOCX) [file pone.0025775.s002.docx]

Table S1. List of *ROBO1* SNPs investigated

| SNP | Location^a^ | BP^b^ | Further Analysis |
| --- | --- | --- | --- |
| rs723766 | 3' UTR | 78,657,774 | No |
| ROBO1_Ser162Ser | exon 3 | 78,987,766 | No |
| rs59931439 | intron 2 | 78,988,130 | No |
| rs1387665 | 5' UTR/promoter | 79,429,811 | Yes |
| rs1546037 | 5' UTR/promoter | 79,434,134 | No |
| rs4510348 | 5' UTR/promoter | 79,438,446 | No |
| rs4680960 | 5' UTR/promoter | 79,449,566 | No |
| rs13076006 | 5' UTR/promoter | 79,452,636 | Yes |
| rs4680962 | 5' UTR/promoter | 79,461,529 | No |
| rs13090440 | 5' UTR/promoter | 79,465,496 | No |
| rs13058752 | 5' UTR/promoter | 79,470,851 | No |
| rs7624099 | 5' UTR/promoter | 79,475,253 | No |
| rs4513416 | 5' UTR/promoter | 79,490,803 | Yes |
| rs4284943 | 5' UTR/promoter | 79,495,754 | No |
| rs9810404 | 5' UTR/promoter | 79,505,072 | Yes |
| rs9853257 | 5' UTR/promoter | 79,524,548 | No |
| rs7640053 | 5' UTR/promoter | 79,531,271 | Yes |
| rs7615149 | 5' UTR/promoter | 79,537,773 | Yes |
| rs7622888 | 5' UTR/promoter | 79,541,896 | Yes |
| rs4264688 | 5' UTR/promoter | 79,546,348 | Yes |
| rs6548621 | 5' UTR/promoter | 79,550,373 | Yes |
| rs7622444 | 5' UTR/promoter | 79,557,927 | Yes |
| rs9832405 | 5' UTR/promoter | 79,559,914 | Yes |
| rs7637338 | 5' UTR/promoter | 79,560,604 | Yes |
| rs6548625 | 5' UTR/promoter | 79,563,987 | Yes |
| rs7626242 | 5' UTR/promoter | 79,567,274 | No |
| rs7623809 | 5' UTR/promoter | 79,568,973 | Yes |
| rs9873952 | 5' UTR/promoter | 79,573,229 | No |
| rs9871445 | 5' UTR/promoter | 79,577,616 | No |
| rs4279056 | 5' UTR/promoter | 79,581,250 | Yes |
| rs9848827 | 5' UTR/promoter | 79,586,304 | No |
| rs9826366 | 5' UTR/promoter | 79,588,523 | Yes |
| rs3923526 | 5' UTR/promoter | 79,784,128 | Yes |
| rs1393370 | 5' UTR/promoter | 79,790,293 | No |
| rs10865579 | 5' UTR/promoter | 79,811,006 | No |
| rs9309833 | 5' UTR/promoter | 79,811,719 | Yes |
| rs7629503 | 5' UTR/promoter | 79,813,292 | Yes |

^a^ Location is based on the isoform b of the ROBO1 gene, whereas all the SNPs are located in intron 3 on the isoform a of the gene.

^b^ Base pair position (BP) was obtained using the NCBI B36 assembly of dbSNP b126
