# Supplementary material for: Influence of ROBO1 and RORA on Risk of Age-Related Macular Degeneration Reveals Genetically Distinct Phenotypes in Disease Pathophysiology
Source: PLoS One. 2011 Oct 6;6(10):e25775. doi: 10.1371/journal.pone.0025775 (PMC3188561; doi:10.1371/journal.pone.0025775)
Supplement: Table S2 — ROBO1 TaqMan Probes. (DOCX) [file pone.0025775.s003.docx]

Table S2. *ROBO1* TaqMan Probes

| **SNP** | **Probe Name** |
| --- | --- |
| rs9832405 | C__11523693_10 |
| rs7622444 | C__29805155_20 |
| rs6548621 | C__11523723_10 |
| rs7615149 | C____409099_10 |
| rs4513416 | C____307534_10 |
| rs59931439 | C__25632225_10 |
| rs1387665 | AHX0JQB |
